# Blood pressure in young Aboriginal and Torres Strait Islander people: analysis of baseline data from a prospective cohort study

**DOI:** 10.5694/mja2.52558

**Published:** 2024-12-11

**Authors:** Berhe W Sahle, Emily Banks, Robyn Williams, Grace Joshy, Garry Jennings, Jonathan C Craig, Nicholas G Larkins, Francine Eades, Rebecca Q Ivers, Sandra Eades

**Affiliations:** ^1^ The University of Melbourne Melbourne VIC; ^2^ Centre for Public Health Data and Policy, National Centre for Epidemiology and Population Health Australian National University Canberra ACT; ^3^ Curtin University Perth WA; ^4^ National Heart Foundation of Australia Canberra ACT; ^5^ Flinders University Adelaide SA; ^6^ Perth Children's Hospital Perth WA; ^7^ The University of Western Australia Perth WA; ^8^ East Metropolitan Health Service Perth WA; ^9^ University of New South Wales Sydney NSW; ^10^ The George Institute for Global Health UNSW Sydney Sydney NSW

**Keywords:** Hypertension, Indigenous health, Risk factors

## Abstract

**Objective:**

To assess the distribution of blood pressure levels and the prevalence of hypertension and pre‐hypertension in young Indigenous people (10–24 years of age).

**Study design:**

Prospective cohort survey study (Next Generation: Youth Wellbeing Study); baseline data analysis.

**Setting, participants:**

Aboriginal and Torres Strait Islander people aged 10–24 years living in regional, remote, and urban communities in Central Australia, Western Australia, and New South Wales; recruitment: March 2018 – March 2020.

**Main outcome measures:**

Blood pressure categorised as normal, pre‐hypertension, or hypertension using the 2017 American Academy of Pediatrics guidelines (10–17 years) or 2017 American College of Cardiology/American Heart Association guidelines (18–24 years); associations of demographic characteristics and health behaviours with hypertension and pre‐hypertension, reported as relative risk ratios (RRRs) with 95% confidence intervals (CIs).

**Results:**

Complete data were available for 771 of 1244 study participants (62%); their mean age was 15.4 years (standard deviation [SD], 3.9 years), 438 were girls or young women (56.8%). Mean systolic blood pressure was 111.2 mmHg (SD, 13.7 mmHg), mean diastolic blood pressure 66.3 mmHg (SD, 11.0 mmHg). Mean systolic blood pressure was higher for male than female participants (mean difference, 6.38 mmHg; 95% CI, 4.60–8.16 mmHg), and it increased by 1.06 mmHg (95% CI, 0.76–1.36 mmHg) per year of age. Mean systolic blood pressure increased by 0.42 mmHg (95% CI, 0.28–0.54 mmHg) and diastolic blood pressure by 0.46 mmHg (95% CI, 0.35–0.57 mmHg) per 1.0 kg/m^2^ increase in body mass index. Ninety‐one participants (11.8%) had blood pressure readings indicating pre‐hypertension, and 148 (19.2%) had hypertension. The risks of pre‐hypertension (RRR, 4.22; 95% CI, 2.52–7.09) and hypertension (RRR, 1.93; 95% CI, 1.27–2.91) were higher for male than female participants; they were greater for people with obesity than for those with BMI values in the normal range (pre‐hypertension: RRR, 2.39 [95% CI, 1.26–4.55]; hypertension: RRR, 3.20 [95% CI, 1.91–5.35]) and for participants aged 16–19 years (pre‐hypertension: 3.44 [95% CI, 1.88–6.32]; hypertension: RRR, 2.15 [95% CI, 1.29–3.59]) or 20–24 years (pre‐hypertension: 4.12 [95% CI, 1.92–8.85]; hypertension: RRR, 4.09 [95% CI, 2.24–7.47]) than for those aged 10–15 years.

**Conclusions:**

Blood pressure was within the normal range for most young Indigenous people in our study, but one in three had elevated blood pressure or hypertension. Community‐level, culturally safe approaches are needed to avoid the early onset of cardiovascular risks, including elevated blood pressure.

**The known**: Cardiovascular disease‐related mortality has significantly declined among Aboriginal and Torres Strait Islander Australians. However, 31% of Indigenous adults have hypertension, the leading cause of avoidable deaths of Indigenous people.

**The new**: About 70% of Indigenous young people from regional, remote, and urban communities had normal blood pressure, but one in eight had elevated blood pressure and one in five hypertension.

**The implications**: Hypertension in Indigenous young people must be prevented to avert cardiovascular disease in later life. Reducing early cardiovascular risks, while avoiding unnecessary medicalisation and deficit‐framing, is critical for promoting cardiovascular health throughout life.

High blood pressure is the leading cause of the global burden of disease because of its high prevalence and strong association with cardiovascular disease (CVD) and other conditions; it is responsible for 8.5 million deaths each year.^1^, ^2^ An estimated 1–3% of adolescents have hypertension.^1^ The World Health Organization has set the target of reducing the prevalence of hypertension by 33% between 2010 and 2030.^3^

The age‐standardised rate of death from CVD among Aboriginal and Torres Strait Islander (Indigenous) people declined by 33% during 2006–2019, and the total age‐standardised burden of disease attributable to hypertension fell by 45% during 2003–2018, from 41 to 23 per 1000 people.^4^ Aboriginal Community Controlled Health Organisations contribute to improving the health and wellbeing of Indigenous people by providing accessible primary health care.^5^, ^6^ However, 31% of Indigenous adults have hypertension, and it is the leading cause of avoidable deaths of Indigenous people.^4^ High blood pressure is also among the seven leading factors that contribute to health gaps between Indigenous and non‐Indigenous Australians.^7^

High blood pressure during childhood persists into adulthood, and is associated with certain subclinical conditions during childhood and young adulthood (eg, heart, kidney, brain damage) and, later in life, with chronic diseases, including heart failure, stroke, and kidney failure, as well as premature death.^8^, ^9^ The risks of hypertension‐related CVD and death increase with younger age of onset.^10^ CVD risk and clinical events develop 15–20 years earlier in Indigenous people than in non‐Indigenous Australians.^11^, ^12^ Studies based on data collected during 2008–11 found that 15.6% of a sample of urban Indigenous children (2–17 years) in New South Wales^13^ and 21.0% of a sample of young people aged 15–24 years in remote Queensland had hypertension.^14^

More recent estimates of rates of early onset high blood pressure in Indigenous young people in regional, urban, and remote communities are not available. Knowing the scale and developmental timing of the onset of high blood pressure in Indigenous young people, however, is crucial for public health programs for reducing the incidence and impact of early CVD risk. Analyses of a nationally representative sample of Indigenous people aged 18–74 years found that substantial proportions of those under 35 years of age are at high risk of CVD (5‐year absolute risk of a primary CVD event greater than 15%).^15^ However, absolute CVD risk assessment is not currently recommended by guidelines for people under 30 years of age.^16^

In this study, we investigated the distribution of blood pressure and the prevalence of hypertension and pre‐hypertension among young Indigenous people (10–24 years of age) living in regional, remote, and urban communities in Central Australia, Western Australia, and New South Wales. We used a strengths‐based approach that emphasises recognising and building upon the inherent strengths and resilience of Indigenous people, fostering a positive and empowering perspective.^17^, ^18^

## Methods

We analysed baseline data from the Next Generation: Youth Wellbeing Study (NextGen study), a prospective cohort study of 1244 Indigenous people aged 10–24 years living in regional, remote, and urban communities in Western Australia, New South Wales, and the Central Australia region of the Northern Territory.^19^ Indigenous research officers recruited participants through community networks and contacts, youth centres, sporting clubs, and youth health services during 1 March 2018 – 31 March 2020. Young people were engaged in the study by trusted adults who were leaders in their communities by Indigenous researchers.^20^ Information was collected in face‐to‐face interviews, clinical assessments, and point‐of‐care tests. We report our findings according to the Strengthening the Reporting of Observational Studies in Epidemiology (STROBE) statement.^21^

Blood pressure was measured on the non‐dominant arm of each participant three times, at two to three minute intervals, with digital automatic blood pressure monitors (HEM‐907, Omron) between 1 March 2018 and 31 March 2020. Participants were asked to remove any clothing that could interfere with measurement. Blood pressure was measured after participants completed a 45–60‐minute self‐administered questionnaire, allowing sufficient time to ensure that no coffee was consumed or cigarettes smoked immediately before blood pressure measurement. Prior to the first measurement, the participant was given at least five minutes to relax, with legs uncrossed, their back and arm supported, and their feet flat on the floor. The correct cuff size was chosen by confirming that the end of the cuff sat within the cuff guide on the non‐dominant arm. The mean value of the second and third readings was used for analyses.

For each participant, we also collected socio‐demographic information (age, sex) and information on health and risk factors (sleep quality; self‐rated health; smoking; fruit, vegetable, and high salt snack intake; physical activity; recreational screen time) in interviews. Indigenous researchers conducted physical (height, weight) and clinical measurements (details: Supporting Information, part 1).

### Blood pressure classification

For participants aged 10–12 years, we used the 2017 American Academy of Pediatrics classification of blood pressure by sex, age, and height percentiles for children under 13 years of age:

- elevated blood pressure: 90th percentile to less than 95th percentile, or 120 mmHg (systolic)/80 mmHg (diastolic) to less than 95th percentile (whichever is lower);
- stage 1 hypertension: 95th percentile to less than 95th percentile +12 mmHg, or 130/80 mmHg to 139/89 mmHg (whichever is lower); and
- stage 2 hypertension: 95th percentile +12 mmHg, or 140/90 mmHg (whichever is lower).^22^

For participants aged 13–17 years, we used the following values:

- elevated blood pressure: 120 mmHg/less than 80 mmHg to 129 mmHg/less than 80 mmHg;
- stage 1 hypertension: 130/80 mmHg to 139/89 mmHg; and
- stage 2 hypertension 140/90 mmHg or greater.^22^

For participants aged 18 years or older, pre‐hypertension and hypertension were defined according to the 2017 American College of Cardiology/American Heart Association guideline recommendations:

- normal blood pressure: less than 120 mmHg (systolic) and less than 80 mmHg (diastolic);
- elevated blood pressure: 120–129 mmHg (systolic) and less than 80 mmHg (diastolic);
- stage 1 hypertension: 130–139 mmHg (systolic) or 80–89 mmHg (diastolic); and
- stage 2 hypertension: 140 mmHg or more (systolic) or 90 mmHg or more (diastolic).^23^

### Data analysis

Participants with systolic blood pressure measurements below 70 mmHg or greater than 270 mmHg, or diastolic blood pressure below 50 mmHg or greater than 150 mmHg, were excluded.^24^ That is, standard cut‐off points for implausible blood pressure values more than five standard deviations from the mean value were applied.^25^

We summarise participant characteristics as numbers and proportions and as means with standard deviations (SDs). We assessed the relationship between participant characteristics and systolic and diastolic blood pressure in multivariate linear regression models. The relationship between body mass index (BMI) and blood pressure was assessed in a multivariate linear regression model with BMI added as a continuous variable. We examined whether the association between BMI and blood pressure differed by sex or age by fitting interaction terms in the regression models.

We estimated relative risk ratios (RRRs) with 95% confidence intervals (CIs) in multinominal logistic regression models to assess associations of demographic characteristics and health behaviours with hypertension and pre‐hypertension. Regression models treating the responses “I don't know,” “I don't remember,” and “Prefer not to say” as separate categories or missing values yielded similar results; we report analyses in which these responses were treated as missing values. Multiple imputation by chained equations (100 estimates) was used to adjust for missing covariate values.

### Indigenous leadership, governance, and engagement

The NextGen study is an Indigenous‐led initiative with substantial Indigenous leadership and engagement. The idea for the study emerged following community consultations in Western and Central Australia aimed at identifying and defining research priorities. Local Indigenous organisations and agencies were engaged from the outset and provided written letters of support for the study. Prior to the commencement of baseline data collection, qualitative interviews and focus group discussions were conducted with young Indigenous people to review the research aims and methodology. This process ensured alignment with local and cultural priorities. Strong connections with local communities and elders were a critical component of engagement with young people in safe community or family environments.^20^ Data collection was primarily conducted by Indigenous research officers. Three Indigenous team members participated in the interviews, data analysis, and preparation of the manuscript for this article.

### Ethics approval

The study was approved by the Central Australian Human Research Ethics Committee (16‐398), the Western Australian Aboriginal Health Ethics Committee (619, 719), the Aboriginal Health and Medical Research Council of NSW Ethics Committee (1255‐17), the Alfred Health Ethics Committee (255‐16), and the University of Melbourne Medicine and Dentistry Human Ethics Sub‐Committee (1851155).

## Results

Of 1244 young people recruited for the NextGen study, three recorded diastolic and systolic blood pressure measurements were available for 822 (66%); 51 participants were excluded because height data were not recorded (needed for computing blood pressure percentiles) or their recorded diastolic blood pressure was less than 50 mmHg (Box 1). The mean age of the 771 included participants (62%) was 15.4 years (SD, 3.9 years; 435 [56.4%] under 16 years of age); 438 were girls or young women (56.8%), and 467 lived in Western Australia (60.6%). Most participants rated their overall health as excellent (142, 19.1%), very good (174, 23.4%), or good (249, 33.5%) (Box 2). About 80% reported consuming at least two servings of fruits and two servings of vegetables per day, and 184 (29.7%) reported consuming high salt snacks no more than once per week.

Box 1 **Selection of Next Generation: Youth Wellbeing (NextGen) study participants for our study of blood pressure in young Aboriginal and Torres Strait Islander people in Western Australia, New South Wales, and Central Australia**

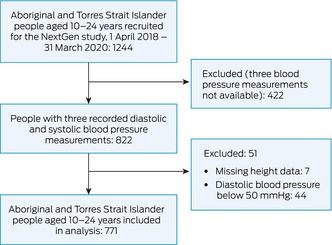

Box 2 **Characteristics of participants in our study of blood pressure in young Aboriginal and Torres Strait Islander people in Western Australia, New South Wales, and Central Australia**

**Table:** 

Characteristic	Number*	Missing data^†^
Participants	771	—
Aboriginal and/or Torres Strait Islander status		—
Aboriginal	753 (97.7%)	
Aboriginal and Torres Strait Islander	17 (2.2%)	
Torres Strait Islander	1 (0.1%)	
Sex (female)	438 (56.8%)	0
Age (years), mean (SD)	15.4 (3.9)	0
10–15	435 (56.4%)	
16–19	203 (26.3%)	
20–24	133 (17.3%)	
Study site		0
Western Australia	467 (60.6%)	
New South Wales	258 (33.5%)	
Central Australia	46 (6.0%)	
Self‐rated health		27 (3.5%)
Excellent	142 (19.1%)	
Very good	174 (23.4%)	
Good	249 (33.5%)	
Fair/poor	140 (18.8%)	
Do not know	39 (5.2%)	
Body mass index (kg/m^2^), mean (SD)^‡^	24.0 (7.5)	40 (5.2%)
Normal	391 (53.5%)	
Overweight	168 (23.0%)	
Obese	172 (23.5%)	
Sleep quality		38 (4.9%)
Very good	280 (38.2%)	
Fairly good	332 (45.3%)	
Fairly bad/very bad	121 (16.5%)	
Serves of fruit per day		18 (2.3%)
0 or 1	82 (10.9%)	
2–4	416 (55.2%)	
5 or more	190 (25.2%)	
Do not remember	65 (8.6%)	
Serves of vegetables per day		17 (2.2%)
0 or 1	82 (10.9%)	
2–4	426 (56.5%)	
5 or more	175 (23.2%)	
Do not remember	71 (9.4%)	
Serves of high salt snacks per week		152 (19.7%)
0 or 1	184 (29.7%)	
2‐3	225 (36.4%)	
4 or more	210 (33.9%)	
Physical activity		0
High^§^	295 (38.3%)	
Low	312 (40.5%)	
Do not remember	164 (21.2%)	
Recreational screen time (hours per day)		48 (6.2%)
Less than two	150 (20.7%)	
Two or more	479 (66.2%)	
Do not remember	94 (13.0%)	
Smoked cigarettes (ever)		25 (3.2%)
No	499 (66.9%)	
Yes	218 (29.2%)	
Prefer not to say	29 (3.9%)	
People smoke inside the house you live in		60 (7.8%)
Never	563 (79.2%)	
Yes	148 (20.8%)	

SD = standard deviation.

* Excludes missing data.

† Includes “Not answered”.

‡ 10–19 years: overweight, body mass index (BMI)‐for‐age *Z*‐score of more than 2; obesity, BMI‐for‐age *Z*‐score more than 1. 19–24 years: normal, 18.5–24.9 kg/m^2^; overweight, 25.0–29.9 kg/m^2^; obesity, 30.0 kg/m^2^ or more.

§ At least three days or 150 minutes of moderate to vigorous physical activity per week.

### Blood pressure

Mean systolic blood pressure was 111.2 mmHg (SD, 13.7 mmHg), the mean diastolic blood pressure 66.3 mmHg (SD, 11.0 mmHg). Mean systolic blood pressure was higher for male than female participants (114.2 [SD, 15.1] *v* 108.9 [SD, 12.2] mmHg); mean diastolic blood pressure was similar for male and female participants (65.3 [SD, 11.9] *v* 67.1 [SD, 10.2] mmHg) (Box 3). Both systolic and diastolic blood pressure increased with age (Box 4) and BMI category (Box 5).

Box 3 **Mean blood pressure of participants in our study in young Aboriginal and Torres Strait Islander people in Western Australia, New South Wales, and Central Australia, by participant characteristics**

**Table:** 

	Blood pressure (mmHg), mean (SD)	
Characteristic	Systolic	Diastolic
All participants	111.2 (13.7)	66.3 (11.0)
Sex		
Male	114.2 (15.1)	65.3 (11.9)
Female	108.9 (12.2)	67.1 (10.2)
Age group (years)		
10–15	107.0 (12.3)	63.8 (10.3)
16–19	114.5 (13.1)	68.3 (11.2)
20–24	119.5 (14.0)	71.5 (10.5)
Study site		
Western Australia	109.9 (13.8)	66.1 (11.1)
New South Wales	112.3 (13.6)	65.9 (11.1)
Central Australia	117.8 (10.8)	70.9 (9.4)
Self‐rated health		
Excellent	108.3 (14.2)	63.7 (10.0)
Very good	110.0 (12.6)	66.0 (11.0)
Good	111.8 (13.5)	66.6 (10.9)
Fair/poor	115.5 (14.3)	69.7 (11.9)
Body mass index category*		
Normal	108.4 (13.1)	63.2 (10.4)
Overweight	111.5 (12.3)	67.0 (10.5)
Obese	117.2 (14.3)	72.5 (10.5)
Sleep quality		
Fairly good	108.7 (12.9)	65.3 (10.8)
Fairly bad/very bad	111.9 (13.9)	66.1 (10.5)
very good	114.8 (13.8)	69.5 (11.3)
Serves of fruit per day		
0 or 1	114.6 (13.0)	69.3 (12.7)
2–4	111.2 (14.2)	66.0 (11.1)
5 or more	109.8 (13.0)	65.9 (10.0)
Serves of vegetables per day		
0 or 1	110.6 (13.1)	65.5 (12.0)
2–4	111.2 (13.7)	66.2 (10.8)
5 or more	110.9 (14.1)	67.1 (10.1)
Serves of high‐salt snacks per week		
0 or 1	110.8 (13.9)	66.4 (11.3)
2–3	110.5 (13.4)	66.0 (10.7)
4 or more	110.8 (13.5)	65.1 (10.5)
Physical activity		
High^†^	111.7 (13.8)	68.2 (11.1)
Low	110.5 (13.6)	64.5 (10.7)
Recreational screen time (hours per day)		
Less than two	109.3 (13.5)	65.2 (11.3)
Two or more	112.0 (13.7)	66.6 (10.9)
Smoked cigarettes (ever)		
Yes	109.4 (13.0)	65.1 (10.8)
No	115.4 (13.6)	68.8 (10.3)

SD = standard deviation.

* 10–19 years: overweight, body mass index (BMI)‐for‐age *Z*‐score of more than 2; obesity, BMI‐for‐age *Z*‐score more than 1. 19–24 years: normal, 18.5–24.9 kg/m^2^; overweight, 25.0–29.9 kg/m^2^; obesity, 30.0 kg/m^2^ or more.

† At least three days or 150 minutes of moderate to vigorous physical activity per week.

Box 4 **Systolic and diastolic blood pressure of young Aboriginal and Torres Strait Islander participants in Western Australia, New South Wales, and Central Australia, by age and sex**

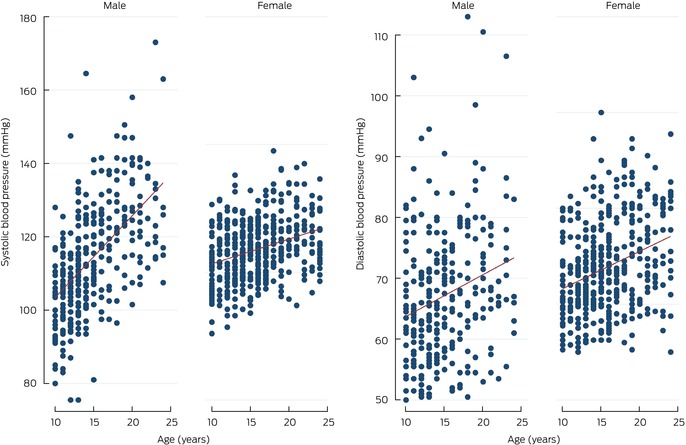

Box 5 **Systolic and diastolic blood pressure of young Aboriginal and Torres Strait Islander participants in Western Australia, New South Wales, and Central Australia, by body mass index**

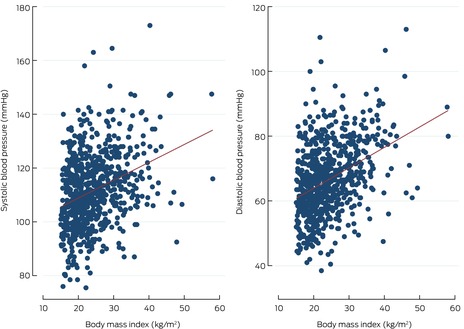

### Relationship between participant characteristics and blood pressure

In the fully adjusted multivariate linear regression model, systolic blood pressure was higher for male than female participants (mean difference, 6.38 mmHg; 95% CI, 4.60–8.16 mmHg), but not diastolic blood pressure. Mean systolic (mean difference, 5.81 mmHg; 95% CI, 2.10–9.51 mmHg) and diastolic blood pressure (4.25 mmHg; 95% CI, 1.15–7.34 mmHg) were higher for participants from Central Australia than for those from Western Australia. Mean systolic blood pressure increased by 0.42 mmHg (95% CI, 0.28–0.54 mmHg) and diastolic blood pressure by 0.46 mmHg (95% CI, 0.35–0.57) per 1.0 kg/m^2^ BMI increase; systolic blood pressure increased by 1.06 mmHg (95% CI, 0.76–1.34 mmHg) per year of age, diastolic blood pressure by 0.42 mmHg (95% CI, 0.17–0.66 mmHg) (Box 6).

Box 6 Systolic and diastolic blood pressure of young Aboriginal and Torres Strait Islander participants in Western Australia, New South Wales, and Central Australia: fully adjusted multivariate linear regression analysis*

**Table:** 

	Coefficient (95% CI)	
Characteristic	Systolic blood pressure	Diastolic blood pressure
Sex		
Female	1	1
Male	**6.38 (4.60 to 8.16)**	–0.87 (–2.35 to 0.61)
Age, per year	**1.06 (0.76 to 1.36)**	**0.42 (0.17 to 0.66)**
Study site		
Western Australia	1	1
New South Wales	0.89 (–1.03 to 2.80)	–1.00 (–2.61 to 0.59)
Central Australia	**5.81 (2.10 to 9.51)**	**4.25 (1.15 to 7.34)**
Self‐rated health		
Excellent	1	1
Very good	0.33 (–2.36 to 3.03)	1.77 (–0.50 to 4.05)
Good	0.82 (–1.79 to 3.43)	1.01 (–1.18 to 3.21)
Fair/poor	0.96 (–2.85 to 4.21)	1.55 (–1.76 to 4.28)
Body mass index, per 1.0 kg/m^2^	**0.42 (0.28 to 0.54)**	**0.46 (0.35 to 0.57)**
Sleep quality		
Very good	1	1
Fairly good	0.89 (–1.17 to 2.95)	–0.97 (–2.70 to 0.74)
Fairly bad/very bad	1.85 (–0.94 to 4.63)	0.85 (–1.51 to 3.21)
Serves of fruit (per day)		
0 or 1	0.47 (–3.04 to 3.99)	0.74 (–2.26 to 3.74)
2–4	–0.65 (–2.91 to 1.59)	–0.87 (–2.77 to 1.02)
5 or more	1	1
Serves of vegetables (per day)		
0 or 1	0.10 (–3.66 to 3.46)	–0.54 (–3.60 to 2.50)
2–4	0.27 (–2.05 to 2.59)	0.08 (–1.88 to 2.04)
5 or more	1	1
Serves of high‐salt snacks per week		
0 or 1	1	1
2–3	–0.76 (–3.21 to 1.70)	–0.15 (–2.28 to 1.97)
4 or more	–0.21 (–2.66 to 2.24)	–1.05 (–3.11 to 1.01)
Physical activity		
High^†^	1	1
Low	–1.31 (–3.33 to 0.72)	1.72 (–0.03 to 3.41)
Recreational screen time (hours per day)		
Less than two	1	1
Two or more	0.64 (–1.64 to 2.92)	0.15 (–1.77 to 2.07)
Smoked cigarettes (ever)	–0.36 (–2.78 to 2.06)	–0.61 (–2.67 to 1.45)

CI = confidence interval.

* Adjusted for sex, age, study site, self‐rated health, body mass index, serves of fruits per day, serves of vegetables per day, serves of high salt snacks per week, physical activity, recreational screentime, and ever smoked cigarettes; multiple imputation of missing data.

† At least three days or 150 minutes of moderate to vigorous physical activity per week.

Bold: statistically significant (95% CI does not include 0).

### Prevalence of pre‐hypertension and hypertension

Ninety‐one participants (11.8%) had blood pressure readings that indicated pre‐hypertension, and 148 (19.2%) had hypertension (stage 1, 117 [15.2%]; stage 2, 31 [4.0%]). The crude prevalence of pre‐hypertension was higher among male than female participants (61 of 333, 18.6% *v* 30 of 438, 6.8%); that of hypertension was similar for both sexes (68, 20.8% *v* 80, 18.0%). The crude prevalence of hypertension among participants with BMI values in the normal range was 12.0% (51 participants); it was higher for participants with values in the overweight (33, 19.6%) or obese range (57, 33.1%) (Box 7).

Box 7 **Blood pressure categories of young Aboriginal and Torres Strait Islander participants in Western Australia, New South Wales, and Central Australia, by participant characteristics**

**Table:** 

Characteristics	Normal	Pre‐hypertension	Hypertension
All participants	532 (69.0%)	91 (11.8%)	148 (19.2%)
Sex			
Female	334 (75.2%)	30 (6.8%)	80 (18.0%)
Male	198 (60.5%)	61 (18.6%)	68 (20.8%)
Age category (years), mean (SD)	14.7 (3.6)	16.5 (3.7)	17.1 (4.4)
10–15	344 (79.1%)	36 (8.3%)	55 (12.6%)
16–19	126 (62.1%)	33 (16.3%)	44 (21.7%)
20–24	62 (46.6%)	22 (16.5%)	49 (36.8%)
Study site			
Western Australia	335 (71.7%)	47 (10.0%)	85 (18.2%)
New South Wales	175 (67.8%)	35 (13.6%)	48 (18.6%)
Central Australia	22 (47.8%)	9 (19.6%)	15 (32.6%)
Self‐rated health			
Excellent	107 (75.3%)	13 (9.1%)	22 (15.5%)
Very good	125 (71.8%)	17 (9.8%)	32 (18.4%)
Good	167 (67.1%)	31 (12.4%)	51 (20.5%)
Fair/poor	81 (57.8%)	22 (15.7%)	37 (26.4%)
Body mass index (kg/m^2^), mean (SD)*	22.6 (6.0)	27.0 (5.9)	28.5 (11.0)
Normal	303 (77.5%)	37 (9.4%)	51 (12.0%)
Overweight	115 (68.4%)	20 (11.9%)	33 (19.6%)
Obese	88 (51.2%)	27 (15.7%)	57 (33.1%)
Sleep quality			
Very good	203 (72.5%)	32 (11.4%)	45 (16.1%)
Fairly good	231 (69.6%)	42 (12.6%)	59 (17.8%)
Fairly bad/very bad	73 (60.3%)	13 (10.7%)	35 (28.9%)
Physical activity			
High^†^	222 (71.1%)	38 (12.2%)	52 (16.7%)
Low	198 (67.1%)	28 (9.5%)	69 (23.4%)
Recreational screen time (hours per day)			
Less than two	105 (70.0%)	17 (11.3%)	28 (18.7%)
Two or more	330 (68.9%)	53 (11.0%)	96 (20.0%)
Smoked cigarettes (ever)			
No	362 (68.0%)	54 (59.3%)	83 (56.0%)
Yes	128 (24.0%)	34 (37.4%)	56 (37.8%)

SD = standard deviation.

* 10–19 years: overweight, body mass index (BMI)‐for‐age *Z*‐score of more than 2; obesity, BMI‐for‐age *Z*‐score more than 1. 19–24 years: normal, 18.5–24.9 kg/m^2^; overweight, 25.0–29.9 kg/m^2^; obesity, 30.0 kg/m^2^ or more.

† At least three days or 150 minutes of moderate to vigorous physical activity per week.

### Relationship between participant characteristics and hypertension

In the fully adjusted multivariate model, the risks of pre‐hypertension (RRR, 4.22; 95% CI, 2.52–7.09) and hypertension (RRR, 1.93; 95% CI, 1.27–2.91) were higher for male than female participants. The risks of pre‐hypertension (RRR, 2.39; 95% CI, 1.26–4.55) and hypertension (RRR, 3.20; 95% CI, 1.91–5.35) were higher for people with obesity than for those with BMI values in the normal range. The risks of pre‐hypertension (RRR, 2.75; 95% CI, 1.09–6.92) and hypertension (RRR, 2.63; 95% CI, 1.21–5.70) were higher for Central Australian than Western Australian participants (Box 8). Similar findings were yielded by minimally adjusted models that included only age, sex, and body weight as covariates (Supporting Information, tables 1 and 2).

Box 8 Pre‐hypertension and hypertension in young Aboriginal and Torres Strait Islander participants in Western Australia, New South Wales, and Central Australia: fully adjusted multivariate linear regression analysis*

**Table:** 

	Relative risk ratio (95% CI)	
Characteristic	Pre‐hypertension	Hypertension
Sex		
Female	1	1
Male	**4.22 (2.52–7.09)**	**1.93 (1.27–2.91)**
Age group (years)		
10–15	1	1
16–19	**3.44 (1.88–6.32)**	**2.15 (1.29–3.59)**
20–24	**4.12 (1.92–8.85)**	**4.09 (2.24–7.47)**
Study site		
Western Australia	1	1
New South Wales	1.19 (0.70–2.03)	0.91 (0.58–1.42)
Central Australia	**2.75 (1.09–6.92)**	**2.63 (1.21–5.70)**
Self‐rated health		
Excellent	1	1
Very good	1.23 (0.54–2.78)	1.32 (0.68–2.55)
Good	1.58 (0.73–3.41)	1.28 (0.68–2.41)
Fair/poor	1.84 (0.74–4.56)	1.28 (0.60–2.71)
Body mass index category^†^		
Normal	1	1
Overweight	1.47 (0.77–2.81)	1.54 (0.91–2.59)
Obese	**2.39 (1.26–4.55)**	**3.20 (1.91–5.35)**
Sleep quality		
Very good	1	1
Fairly good	0.85 (0.48–1.50)	0.82 (0.50–1.33)
Fairly bad/very bad	0.70 (0.31–1.56)	1.39 (0.76–2.54)
Serves of fruit		
0 or 1	0.66 (0.24–1.83)	1.10 (0.50–2.40)
2–4	0.93 (0.48–1.80)	0.94 (0.55–1.60)
5 or more	1	1
Serves of vegetables		
0 or 1	1.56 (0.58–4.20)	0.75 (0.31–1.82)
2–4	1.09 (0.55–2.16)	0.97 (0.57–1.64)
5 or more	1	1
Serves of high‐salt snacks		
0 or 1	1	1
2–3	0.51 (0.26–0.99)	0.78 (0.44–1.35)
4 or more	0.55 (0.29–1.06)	0.67 (0.37–1.19)
Physical activity		
High^‡^	1	1
Low	0.62 (0.34– 1.14)	1.05 (0.66–1.67)
Recreational screen time (hours per day)		
Less than two	1	1
Two or more	1.07 (0.56–2.04)	1.03 (0.60–1.76)
Smoked cigarettes (ever)		
Yes	1	1
No	0.96 (0.51–1.80)	0.95 (0.56–1.62)

CI = confidence interval.

* Adjusted for sex, age, study site, self‐rated health, body mass index, serves of fruits per day, serves of vegetables per day, serves of high‐salt snacks per week, physical activity, recreational screentime, and ever smoked cigarettes; multiple imputation of missing data.

† 10–19 years: overweight, body mass index (BMI)‐for‐age *Z*‐score of more than 2; obesity, BMI‐for‐age *Z*‐score more than 1. 19–24 years: normal, 18.5–24.9 kg/m^2^; overweight, 25.0–29.9 kg/m^2^; obesity, 30.0 kg/m^2^ or more.

‡ At least three days or 150 minutes of moderate to vigorous physical activity per week.

Bold: statistically significant (95% CI does not include 1).

## Discussion

We found that blood pressure was within the normal range for 69% of our sample of young Indigenous people in regional, remote, and urban communities. However, blood pressure exceeded the threshold for pre‐hypertension in 11.8% of participants, and that for hypertension in 19.2%. The risks of pre‐hypertension and hypertension were two to four times as high for participants aged 15–24 years as for those aged 10–15 years; 36.8% of participants aged 20–24 years had hypertension. The risk of pre‐hypertension was four times as high for male participants as for female participants, and the risk of hypertension twice as high. The risk of hypertension was three times as high for young people with obesity as for those with BMI values within the normal range.

Our key finding is that more than two‐thirds of Indigenous young people had blood pressure within the normal range, but the prevalence of pre‐hypertension and hypertension were both high. Given the morbidity and mortality risks associated with the early onset of elevated blood pressure, this finding requires further attention. Prevalence in our study was similar to values reported by an earlier study (2008–2011) of Indigenous children aged 2–17 years: 12.3% for pre‐hypertension and 15.6% for hypertension.^13^ Another study, based on data from a screening program in eleven remote north Queensland communities, found a hypertension prevalence of 21% among Indigenous people aged 15–24 years.^14^ A prospective, population‐based cohort study of Indigenous children attending public primary schools across urban, regional, and remote areas of New South Wales found that the prevalence of systolic hypertension rose from 7.2% at baseline (mean age, 10.5 years) to 11.1% six years (mean age, 14.1 years) and 15.4% eight years later (mean age: 18.3 years); the prevalence of diastolic hypertension during the same period was 2–3%.^26^ A cross‐sectional study of 158 young people aged 5–17 years in the Torres Strait region found that 27% had hypertension.^27^

Consistent with other studies of Indigenous children and young people,^13^, ^14^ we found that obesity is associated with pre‐hypertension and hypertension. One study of urban Indigenous children found a 0.8 mmHg increase in systolic blood pressure per 1.0 kg/m^2^ increase in BMI,^13^ similar to the 0.5 mmHg increase we found.

Some factors that contribute to poor cardiovascular health have been improving for Indigenous people in recent years. The proportion of Indigenous people who have never smoked increased from 49% in 2005 to 70% in 2017.^28^ Indigenous children are physically more active, although activity levels tend to drop during the teenage years, especially among young women.^29^, ^30^ However, overweight and obesity rates are increasing among both Indigenous and non‐Indigenous Australians, possibly contributing to the high prevalence of hypertension. Further, as 12.0% of participants in our study with normal BMI had hypertension, other factors probably contribute to high rates of elevated blood pressure. These could include the ongoing impact of colonisation on Indigenous communities and culture, broader socio‐cultural determinants, and experience of racism and discrimination.^31^, ^32^ Low birthweight, an independent predictor of higher blood pressure, is more frequent among Indigenous than non‐Indigenous people, but its rate has declined;^33^ nine of ten Indigenous babies born in 2019 were of healthy birthweight.^4^ The participants in our study were born between 1994 and 2010, when the prevalence of low birthweight may have been higher, potentially increasing the risk of elevated blood pressure.^34^

### Implications

The high prevalence of elevated blood pressure among young Indigenous people in our study has implications for reducing Indigenous health inequities throughout life. Elevated blood pressure in childhood or adolescence is associated with higher CVD morbidity and CVD‐related mortality during adulthood.^35^, ^36^, ^37^ A multi‐country prospective cohort study recently confirmed the importance of blood pressure as a predictor of future cardiovascular events; each standard deviation increase in childhood systolic blood pressure (age 3–19 years) was associated with a 33% increase in the risk of fatal cardiovascular events in adulthood.^35^ The Framingham Heart Study found that the early onset of hypertension (before the age of 45 years) was associated with a considerably greater cumulative risk of premature cardiovascular mortality and target end organ damage than late onset hypertension.^38^, ^39^ Reducing the rate of early onset of CVD risks, including elevated blood pressure, will therefore contribute to the continuing decline in CVD mortality among Indigenous people.

The high prevalence of hypertension and pre‐hypertension in young Indigenous people suggests that Indigenous communities should be supported in providing culturally safe and supportive environments that enable young people to maintain their health and stay connected to their traditions, values, and cultures. The National Aboriginal and Torres Strait Islander Health Plan emphasises the centrality of culture in the health of Indigenous people and the rights of individuals to safe, healthy, empowered lives.^40^ This approach is aligned with the National Preventive Health Strategy recommendation to integrate cultural determinants of health into preventive health initiatives for reducing health inequalities between Indigenous and non‐Indigenous people.^41^ Young people who have a sense of community connection, belonging, and access to sports facilities free of racism and discrimination tend to be more physically active, which is linked with lower risk of overweight and obesity and elevated blood pressure.^42^ Previous Next Generation cohort studies found that more physically active young people have lower BMI values.^30^ In this study, we found that young people with lower BMI values are more likely to have lower blood pressure. Young people who report more youth programs in their communities are more likely to be physically active.^29^ Supporting community‐level sport and other social and recreational programs for young people is essential for reducing population blood pressure levels and other cardiovascular risk factors. Primary care alone is insufficient to prevent hypertension in Indigenous young people.

### Limitations

While our study sample was not representative of all young Indigenous Australians, some characteristics of the Next Generation participants, including BMI distribution, the proportion with no history of smoking, physical activity levels, and self‐rated health, are similar to those reported for Indigenous young people at the national level.^4^ Our finding that many young Indigenous people had elevated blood pressure is consistent with those of other studies, and our identification of risk factors for high blood pressure was based on internal comparisons and is therefore likely to be generalisable to other young Indigenous Australians. Self‐reported data for variables such as diet, physical activity, and smoking can be influenced by recall and social desirability biases, potentially leading to misreporting. Information on pregnancy status and chronic conditions that can affect blood pressure levels was not collected, nor was information about antihypertensive medication use, so that we could not determine whether blood pressure was controlled in participants with hypertension. In our study, blood pressure was measured on a single occasion. The prevalence of pre‐hypertension and hypertension would probably be lower if blood pressure were measured on several separate occasions, as recommended in clinical practice guidelines for hypertension diagnosis.^43^ For example, a meta‐analysis of 21 studies including a total of 179 561 participants aged 3–20 years found pooled hypertension prevalence rates of 12% at first visits, 5.6% at second visits, and 2.7% at third visits.^44^

### Conclusion

While most participants had blood pressure within the normal range, hypertension and pre‐hypertension were alarmingly frequent among the young Indigenous Australians from regional, remote, and urban communities in our study. The risks of hypertension and pre‐hypertension increased with age and BMI category, and were higher for male than female participants. Culturally safe approaches to assessing blood pressure and associated risk factors in Indigenous young people are important for preventing the early development of CVD risk and promoting cardiovascular health throughout life.

## Supporting information


Supplementary methods and results
