# Supplementary material for: Blood pressure in young Aboriginal and Torres Strait Islander people: analysis of baseline data from a prospective cohort study
Source: Med J Aust. 2024 Dec 11;222(2):91–101. doi: 10.5694/mja2.52558 (PMC11787810; doi:10.5694/mja2.52558)
Supplement: Supplementary file 1 — Supplementary methods and results [file MJA2-222-91-s001.pdf]

MJA
The Medical Journal of Australia

Supporting Information

Supplementary methods and results

This appendix was part of the submitted manuscript and has been peer reviewed.
It is posted as supplied by the authors.

Appendix to: Sahle BW, Banks E, Williams R, et al. Blood pressure in young Aboriginal and Torres Strait Islander people: analysis of baseline data from a prospective cohort study. *Med J Aust* 2025; doi: 10.5694/mja2.52558.

Supplementary methods

Additional details on measurement, and definition of study variables

Measurements: Participants provided information on sociodemographic information and on a range of health and risk factors at interview. These included age (and date of birth), gender, sleep quality, self-rated health, smoking, and serves of fruits and vegetables, physical activity and recreational screen time. Participants were asked how many days and hours in total, outside of school hours, they exercised or played sports or games that caused them to sweat and breathe heavily (e.g., basketball, netball, football, biking, running). Consistent with a previous study (1) and physical activity guidelines, we defined ≥3 days or ≥150 minutes of moderate to vigorous physical activity physical per week as a "high"(2).

Recreational screen time was assessed by asking how many hours a day on average participants usually spend viewing screens, including TV, computer, iPad/tablet or phone. The response choices were categorised into less than 2 hours per day and 2 or more hours per day (2). The number of serves of fruit and vegetables each day was grouped into ≤1 serve, 2-4 serves, and 5 or more serves per day (3).

Indigenous researchers conducted physical and clinical measurements. Comprehensive training was provided to all data collectors prior to data collection to ensure measurement consistency. Weight was measured in kilograms and height in centimetres using a digital floor scale placed on a firm surface or board with the participant dressed in light clothing with shoes off.

Body mass index classification: The World Health Organization age-appropriate classifications for body mass index (BMI) were used (4). In those aged 10-19 years old, overweight and obesity were defined as BMI-for-age Z-score of more than 2 and BMI-for-age Z-score more than 1, respectively. In those aged 19 years and older, the adult WHO classification for normal weight (18.5–24.9 kg/m^2), overweight (25.0–29.9 kg/m^2) and obesity (≥30.0 kg/m^2) was used.

References

1. Macniven R, McKay CD, Graham S, et al. Social and behavioural correlates of high physical activity levels among aboriginal adolescent participants of the Next Generation: Youth Wellbeing Study. Int J Environ Res Public Health 2023; 20: 3738.

2. Department of Health and Aged Care. Physical activity and exercise guidelines for all Australians. Archived: https://www.health.gov.au/topics/physical-activity-and-exercise/physical-activity-and-exercise-guidelines-for-all-australians (viewed Nov 2024).

3. National Health and Medical Research Council. Australian dietary guidelines. 2013. https://www.eatforhealth.gov.au/guidelines (accessed 27 June 2023).

4. de Onis M, Onyango AW, Borghi E, et al. Development of a WHO growth reference for school-aged children and adolescents. Bull World Health Organ 2007; 85: 660-667.

Supplementary results

Table 1. Systolic and diastolic blood pressure of young Aboriginal and Torres Strait Islander participants in Western Australia, and New South Wales, and central Australia: minimally adjusted multivariate linear regression analysis*

	**Coefficient (95% CI)**	
**Characteristic**	**Systolic blood pressure**	**Diastolic blood pressure**
Sex (male *v* female participants)	6.80 (5.06–8.54)	-0.83 (-2.30 to 0.64)
Age, per year	1.16 (0.92–1.40)	0.53 (0.32–0.73)
Body mass index, per 1.0 kg/m^2	0.42 (0.29–0.54)	0.48 (0.37–0.58)

CI = confidence interval.

* Adjusted for age, sex, and body mass index; multiple imputation of missing data.

Table 2. Pre-hypertension and hypertension in young Aboriginal and Torres Strait Islander participants in Western Australia, and New South Wales, and central Australia: minimally adjusted multivariate linear regression analysis*

	**Relative risk ratio (95% CI)**	
**Characteristic**	**Pre-hypertension**	**Hypertension**
Sex (male *v* female participants))	**4.57 (2.77–7.54)**	**1.93 (1.27–2.91)**
Age group (years)		
10-15	1	1
16-19	**3.13 (1.82–5.40)**	**2.28 (1.43–3.64)**
20-24	**3.70 (1.97–6.96)**	**4.38 (2.67–7.20)**
Body mass index (category)		
Normal	1	1
Overweight	1.34 (0.73–2.48)	1.57 (0.95–2.60)
Obese	**2.46 (1.38–4.39)**	**3.24 (2.03–5.19)**

CI = confidence interval.

* Adjusted for age group, sex, and body mass index categories; multiple imputation of missing data.
